# Quantitative Near-Infrared Imaging of Platelets in Platelet-Rich Fibrin (PRF) Matrices: Comparative Analysis of Bio-PRF, Leukocyte-Rich PRF, Advanced-PRF and Concentrated Growth Factors

**DOI:** 10.3390/ijms21124426

**Published:** 2020-06-22

**Authors:** Hachidai Aizawa, Tetsuhiro Tsujino, Taisuke Watanabe, Kazushige Isobe, Yutaka Kitamura, Atsushi Sato, Sadahiro Yamaguchi, Hajime Okudera, Kazuhiro Okuda, Tomoyuki Kawase

**Affiliations:** 1 Tokyo Plastic Dental Society, Kita-ku, Tokyo 114-0002, Japan; sarusaru@mx6.mesh.ne.jp (H.A.); tetsudds@gmail.com (T.T.); watatai@mui.biglobe.ne.jp (T.W.); kaz-iso@tc4.so-net.ne.jp (K.I.); shinshu-osic@mbn.nifty.com (Y.K.); atu.net@nifty.com (A.S.); y-sada@mwd.biglobe.ne.jp (S.Y.); okudera@carrot.ocn.ne.jp (H.O.); 2 Division of Periodontology, Institute of Medicine and Dentistry, Niigata University, Niigata 951-8514, Japan; okuda@dent.niigata-u.ac.jp; 3 Division of Oral Bioengineering, Institute of Medicine and Dentistry, Niigata University, Niigata 951-8514, Japan

**Keywords:** near-infrared imaging, platelets, distribution, platelet-rich fibrin

## Abstract

Platelet-rich fibrin (PRF) is a fibrin matrix enriched with platelets. The PRF matrix is thought to form a steep gradient of platelet density around the region corresponding to the buffy coat in anticoagulated blood samples. However, this phenomenon has not yet been proven. To visualize platelet distribution in PRF in a non-invasive manner, we utilized near-infrared (NIR) imaging technology. In this study, four types of PRF matrices, bio-PRF, advanced-PRF (A-PRF), leukocyte-rich PRF (L-PRF), and concentrated growth factors (CGF) were compared. Blood samples collected from healthy, non-smoking volunteers were immediately centrifuged using four different protocols in glass tubes. The fixed PRF matrices were sagittally divided into two equal parts, and subjected to modified immunohistochemical examination. After probing with NIR dye-conjugated secondary antibody, the CD41^+^ platelets were visualized using an NIR imager. In L-PRF and CGF, platelets were distributed mainly on and below the distal surface, while in bio-PRF and A-PRF, platelet distribution was widespread and homogenous. Among three regions of the PRF matrices (upper, middle, and lower), no significant differences were observed. These findings suggest that platelets aggregate on polymerizing fibrin fibers and float up as a PRF matrix into the plasma fraction, amending the current “gradient” theory of platelet distribution.

## 1. Introduction

Platelet-rich fibrin (PRF), as its name implies, is a fibrin matrix enriched with platelets and their growth factors [1]. This characteristic constitution has led PRF to be widely used in regenerative medicine. However, the concentration and distribution of platelets remain unclear. Platelet counts are commonly evaluated using the “subtraction” method [2], which directly determines platelet counts in soluble fractions, e.g., PRF exudate fraction, and indirectly evaluates platelet counts in insoluble PRF matrices. In 2018, we developed a novel method that directly determines platelet counts via digestion using tissue plasminogen activator and demonstrated its validity and accuracy in comparison with those of the subtraction method [3]. To date, micrographic examination methods using scanning electron microscopy (SEM) and immunohistochemical techniques have been the only practical options for evaluating platelet distribution [4,5,6,7]. However, the data obtained using these methods are often too fragmented to adequately evaluate platelet distribution in three-dimensional (3D) structures by reconstruction.

In recent years, efforts to modify the original Chouroun’s method for leukocyte and platelet-rich fibrin (L-PRF) [8] have led to the development of several PRF derivatives, including concentrated growth factors (CGF) [9] and advanced-PRF (A-PRF) [6]. More recently, this has led to the development of another PRF derivative, bio-PRF [10]. According to our understanding, the main goals of these modifications have been to: (1) form mechanically tough fibrin matrices using thicker and well-crosslinked fibrin fibers; (2) improve the capacity for growth factor retention and release; (3) exclude (or include) more leukocytes. To this end, various centrifugal forces (speed) and time frames have been trialed. Furthermore, the types of centrifuges and blood collection tubes have also been optimized. However, the commercial interests of most vendors of equipment dictate branding their own special products, including centrifuges and related supplies, to beat their competitors. As a result, equipment produced by different brands is not often found in one laboratory, which in turn hinders the ability for the comparative analysis or characterization of individual PRF derivatives from a biomedical point of view. 

Regardless of the protocol used, all PRF derivatives are thought to fractionate platelets in a similar manner depending on the specific gravity of the matrix. However, there is a lack of convincing evidence for this phenomenon. In a previous study [11], we used near-infrared (NIR) imaging technology to visualize the platelets in a PRF matrix. NIR rays (with a wavelength of approximately 750 to 2500 nm; wavelengths between 750 to 900 nm are used for clinical imaging) are highly transmittable in living organisms and are often used in animal experiments for non-invasive observation and in surgical navigation systems in clinical settings. Using NIR, we optimized the procedure of antibody probing and washing and successfully visualized platelet distribution [11]. Despite using the same centrifugation protocol, the platelet distribution of a CGF matrix prepared in glass tubes was found to be visibly distinguishable from that of a CGF matrix prepared in silica-coated plastic tubes.

Interestingly, the distribution of platelets did not depend on their specific gravity in the CGF matrix. However, it is worth mentioning that previous SEM examinations of the surface of CGF (PRF) matrices have demonstrated a steep gravity-dependent gradation [7]. However, this may be due to the limitation of this visualization modality. In fact, in a previous study using immunohistochemical examination [12], platelets were found to also be present in the upper regions of a CGF matrix prepared using a programmed fast-spin protocol. In this study, we quantitatively and qualitatively re-evaluated the distribution of platelets in different PRF matrices using a non-invasive NIR imaging method and comparatively analyzed four types of PRF matrices.

## 2. Results

**Figure 1:**
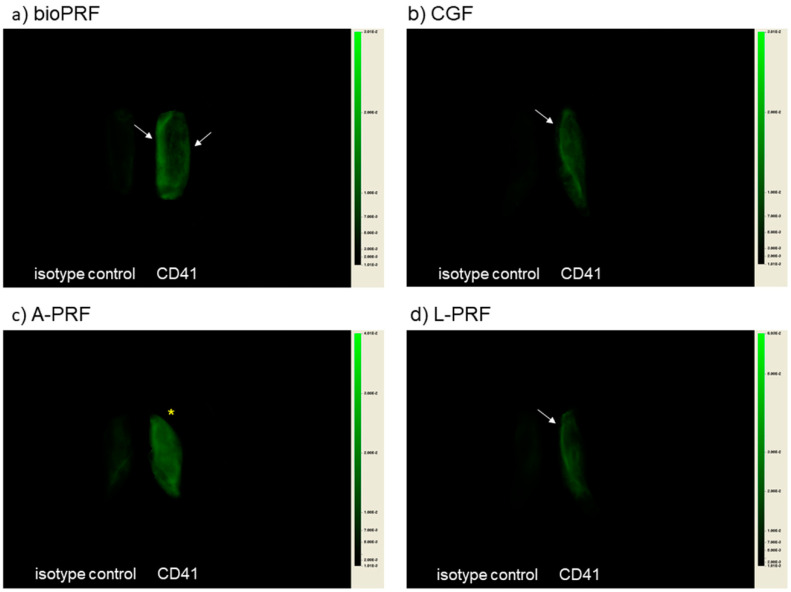
NIR images of compressed half PRF matrices: (**a**) bio-PRF (horizontal, fast spin); (**b**) Concentrated growth factors (CGF) (fixed angle, fast programmed spin); (**c**) A-PRF (fixed angle, slow spin); (**d**) L-PRF (fixed angle, fast spin). White arrows represent platelet localization. An asterisk denotes homogeneous platelet distribution.

The platelet distributions visualized by NIR imaging are shown in Figure 1. Two separate pieces of PRF matrices were placed side-by-side (isotype control image (left) and CD41^+^ image (right)). In the bio-PRF matrix, the platelets were symmetrically distributed mainly in the peripheral regions along the long axis. In the CGF and the L-PRF matrices, the platelets were found to be accumulated mainly in the distal peripheral regions. In contrast, in the A-PRF matrix prepared by slow spin, the platelets were distributed nearly homogeneously.

**Figure 2:**
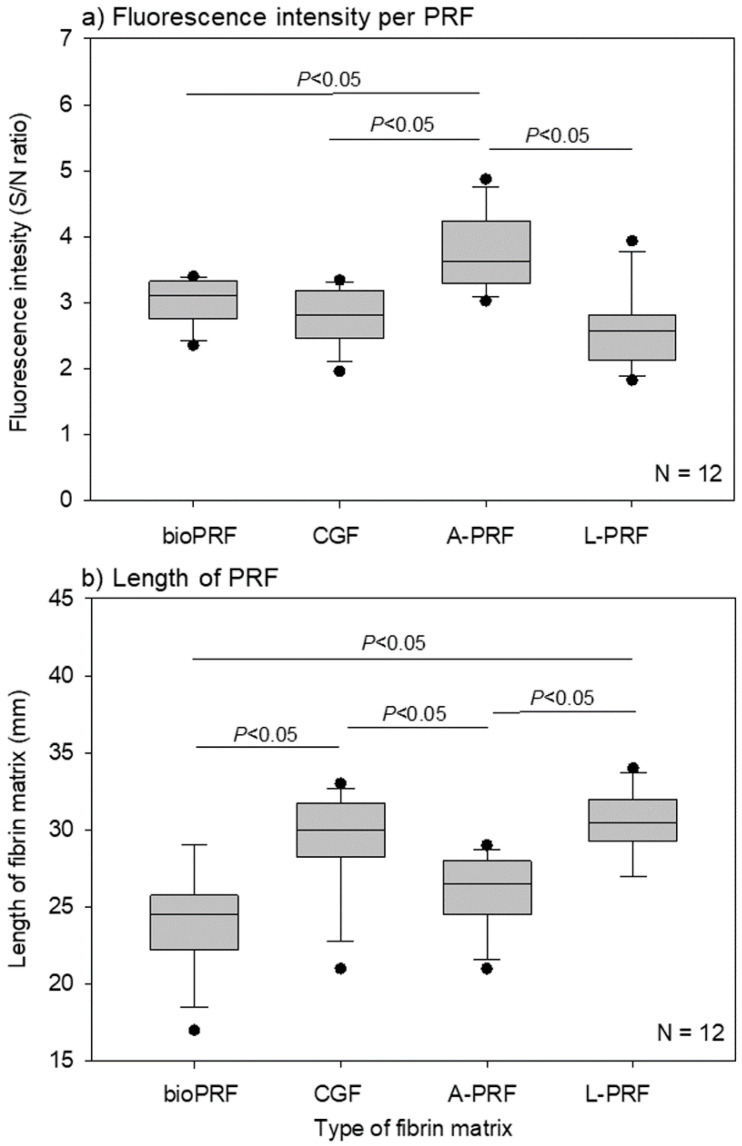
(**a**) Specific fluorescence intensities of individual half platelet-rich fibrin (PRF) matrices. The data are expressed as the CD41^+^-isotype control ratio. *n* = 12. (**b**) The lengths of individual PRF matrices. *n* = 12.

The quantitative determination of the fluorescence intensity of the individual PRF matrices is shown in Figure 2a. These fluorescence intensities represent the total counts of non-activated and activated platelets. The A-PRF matrix was found to contain the highest number of platelets.

The apparent lengths of the individual PRF matrices are shown in Figure 2b. As the shape of the bio-PRF matrix was different from those of the other matrices, its length could not be used for comparison. However, among the PRF matrices prepared using fixed angle rotors, the A-PRF matrix prepared by slow spin was found to be shorter than the L-PRF and CGF matrices.

**Figure 3:**
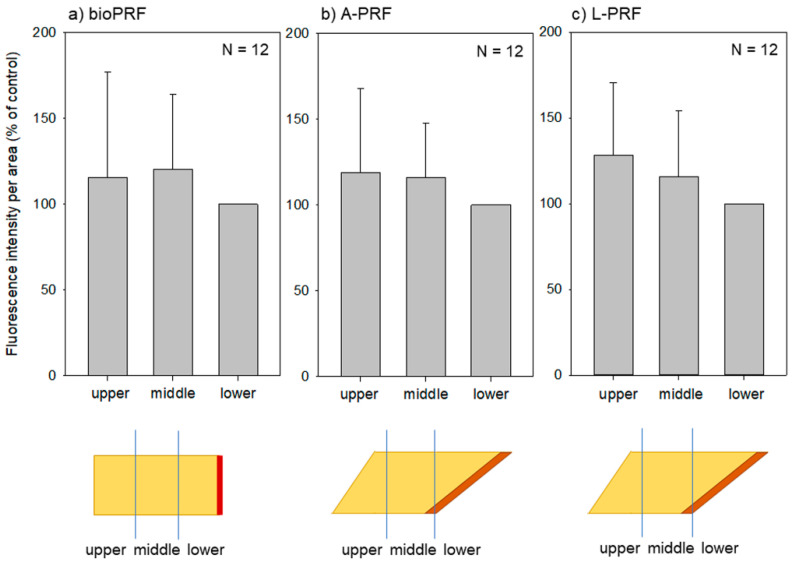
Fluorescence intensities of three regions (upper, middle, and lower) of individual PRF matrices: (**a**) bio-PRF (horizontal, fast spin); (**b**) A-PRF (fixed angle, slow spin); (**c**) L-PRF (fixed angle, fast spin). No significant differences were found among these regions. *n* = 12.

A comparison of the fluorescence intensities of the three regions (upper, middle, and lower) is provided in Figure 3. In the bio-PRF matrix, no significant differences were observed among these regions, and similar observations were made for both the A-PRF and L-PRF matrices.

**Figure 4:**
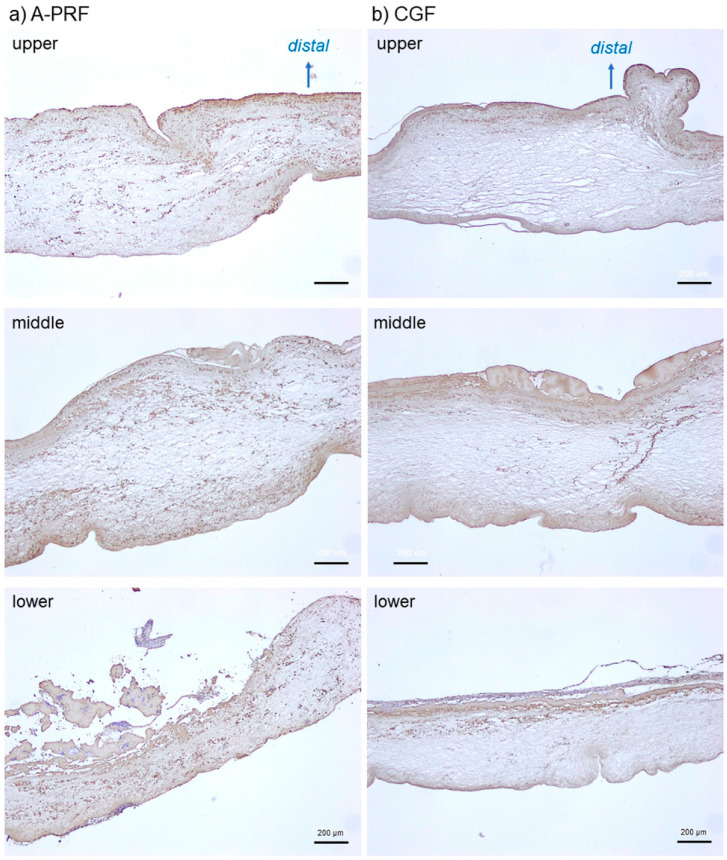
Representative photomicrographs of conventional immunohistochemically-stained sections showing platelet distribution in A-PRF prepared by slow spin (**a**) and CGF prepared by fast spin. (**b**) Brown particles represent platelets. Scale bar, 200 μm.

To confirm the validity of the imaging data, we examined the platelet distribution using conventional immunohistochemical methods using paraffin sections. The representative platelet distributions in the three regions of the A-PRF and CGF matrices are shown in Figure 4. As observed through NIR imaging, the platelets appeared to be distributed nearly homogeneously in the A-PRF matrix prepared by slow spin. In the CGF matrix, in contrast, the platelets appeared to be localized mainly in the distal peripheral regions of all three regions. In addition, in both matrices, the platelets could also be detected in the upper region as observed in the other two regions.

## 3. Discussion

### 3.1. Advantages and Limitations of Our Imaging Method

As described in our previous study [11], although our imaging method was not capable of quantitatively examining the 3D structure of the PRF matrix, the main advantage of using the compressed PRF membrane was the quantitative and qualitative evaluation of platelet distribution without the need for destruction or sectioning. Although the non-specific binding of the antibodies may raise concerns, we overcame this shortcoming by making several technical modifications, including interrupted gentle mixing during fixation, prolonged incubations in PBS and blocking solution, short-term incubations with secondary antibody, and extensive washings using a vortex mixer at each interval.

Furthermore, the samples were divided into two equal parts, one part for specific binding and the other for non-specific binding, to calculate the signal-to-noise (S/N) ratio. This modification resulted in a remarkable improvement in specificity. 

With regards to other shortcomings, the NIR imager was originally designed for animal observation. As such, its resolution (85 μm) was not high enough to observe individual platelets (ϕ1–3 μm). However, since the length of the whole PRF matrix, when compressed, was 25–40 mm, it was slightly too large to observe without image pasting using conventional microscopes. Moreover, it is difficult to evaluate the fluorescence intensity of a subject by image analysis using fluorescence images comprised of photos pasted together without significant distortion. Thus, we accepted the trade-off between the resolution and the one-shot observation.

### 3.2. How Are Platelets Distributed in PRF Matrices?

To date, it remains generally accepted that the distribution of platelets in insoluble PRF matrices depends on their specific gravity, as observed in liquid platelet-rich plasma (PRP) preparations. However, this is inconsistent with the data. If the number of platelets in PRF matrices was similar to that in single-spin-prepared PRP preparations, it would account for roughly 40–70% of the total platelet counts in whole-blood samples. However, several studies have reported that over 90% of platelets are included in PRF matrices by the subtraction method [2,13,14,15].

The main finding of the present study is that platelet distribution in PRF matrices is not gravity-dependent. In the case of centrifuges equipped with fixed angle rotors, the platelet distribution was found to be mainly influenced by the level and direction of the centrifugal force and the distance from the glass surface but not by the angulation level. When centrifuged by slow spin, the platelets were distributed homogeneously. When centrifuged by fast spin, the platelets were distributed on and below the surface, facing the distal inner wall of glass tubes, i.e., that which had received the centrifugal force. In addition, the programmed changes in centrifugal speed did not appear to substantially alter the platelet distribution. On the contrary, in the case of horizontal centrifuges equipped with swing rotors, the platelets were distributed almost homogenously, even at relatively fast centrifugation speeds.

### 3.3. What Does the Difference in Platelet Distribution Imply?

**Figure 5:**
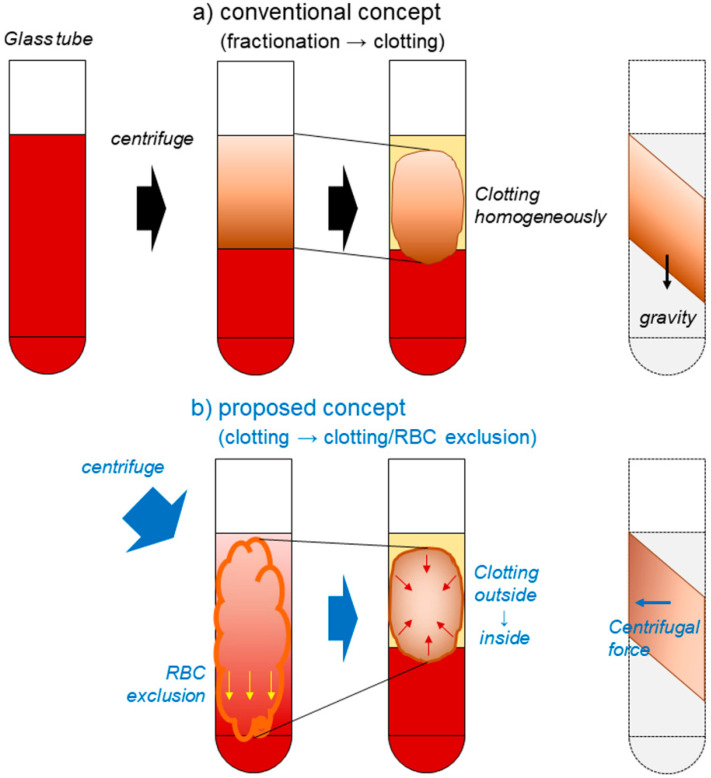
Schematic representation of PRF formation during centrifugation and the resulting platelet accumulation in specific regions of PRF matrices. (**a**) According to the conventional and widely-accepted concept, specific gravity-dependent fractionation precedes coagulation almost homogenously. Thus, the gradation of platelet density is preserved in PRF matrices. (**b**) In our proposed concept, coagulation occurs from the outside, a little earlier than fractionation. Thus, platelets entrapped loosely in gradually clotting PRF matrices are accumulated in the distal peripheral region. This proposed mechanism is consistent with our observations.

During coagulation, the majority of platelets aggregate on fibrin fibers during the process of fibrin formation. As illustrated in Figure 5, the platelets entrapped within gradually coagulating fibrin clots are then fractionated by the specific gravity of the fibrin clot‒platelet complexes, rather than by the platelets alone. Furthermore, fibrin polymerization is triggered by the contact between coagulation factor XII and the glass surface [16]. Due to the activation of platelets on the glass surface, especially on the distal side of the inner wall, fibrin formation is asymmetrical, according to the centrifugal force. Therefore, it is plausible that platelets can be accumulated synergistically by centrifugal force and simultaneous coagulation on the distal side, in the case of the fast spin.

By contrast, the conventional and widely-accepted notion of PRF formation states that platelets are initially fractionated along with other blood cells depending on their specific gravities. Subsequently, coagulation leads to the entrapment of platelets. Therefore, in this case, platelets must accumulate on the interface between the RBC fraction and the PRF matrix, which corresponds to the “buffy coat” in the PRP preparation. Our findings do not support this concept.

### 3.4. What Does the Platelet Distribution Influence?

In several previous studies [3,7,17,18], we have developed techniques and standards to ensure the quality of PRP and PRF during their preparation. If carried out aseptically, as designed, the remaining criteria for quality assurance during the preparation of the matrices include the efficacy and potency of PRF matrices in clinical applications. Since growth factors are served by platelets upon activation and because thrombin and other coagulation factors are also stored by platelets, to guarantee the quality of each preparation and predict each resulting clinical outcome, platelet quantity and quality must be evaluated.

In a previous study [19], we observed that major growth factors (e.g., TGFβ1, PDGF-BB, and VEGF) accumulate on and below the surface of CGF matrices, facing the proximal side of the inner wall, that is, the opposite side of platelet accumulation. These findings suggest that both platelets, which retain growth factors unless fully activated, and the released growth factors are localized to the surface and peripheral regions of the matrices. Thus, the fact that fibrin fibers are digested from the surface regions of implantation sites implies that growth factors are released within relatively short periods of time. The capacity of this release is too low to continuously influence the surrounding tissues and cells.

To evaluate the distribution of growth factors, direct imaging of growth factors was used to obtain more convincing data. In the case of platelet detection, imaging methods require monoclonal antibodies with a high specificity and affinity; however, we have not yet found the appropriate antibodies for the growth factors or other major components. Currently, this represents an additional limitation of this method.

### 3.5. Clinical Relevance of These Findings

To improve the quality of PRF matrices, it is important to establish standardized preparation protocols. However, even during the preparation of PRF matrices, which are less affected by the skills of the operators, biases can arise due to practical constraints and individual donors. Therefore, these need to be minimized. For example, although silica-coated plastic tubes can be used to prepare macroscopically similar PRF matrices as glass tubes, the former PRF matrices are distinguishable from the latter in terms of platelet distribution and contamination with health-hazardous silica microparticles [20,21]. Thus, we propose the necessity for the “inspection of all products.” In other words, advanced cell-medicinal products should all be inspected prior to their clinical application. By contrast, in the case of PRF matrices, which can be prepared aseptically and quickly, inspection is performed to evaluate the components that influence their potency, rather than for the identification of negative criteria that may pose health hazards. As such, inspection may be performed prior to clinical use or the inspection data may be obtained after clinical use. The data can be useful for randomized clinical trials and subsequent meta-analyses to establish evidence-based medication for platelet concentrates.

To date, we have developed several methods for the quality assurance of PRF preparation. The platelet quantity and level of aggregation activity can be tested at the point of care, only requiring short periods of time. In contrast, NIR imaging, such as immunological detection, requires at least two days. However, this is almost as long as the time for paraffin section-based immunohistochemical examination. Thus, regardless of the protocols and devices used for PRF preparation, we expect NIR imaging to provide a potent strategy for evaluating the quality of PRF matrices in the near future.

## 4. Materials and Methods 

### 4.1. Preparation of PRF Matrices

Blood samples were collected from six healthy, non-smoking male and female volunteers aged 31 to 71 years. Depending on the purpose of each experiment, the minimum essential number of donors was randomly selected for sample collection. In the experiments using a CGF matrix prepared from whole-blood samples, blood samples were collected on two occasions at one-week intervals. The number of samples used is indicated in the legend corresponding to each figure. The study design and consent forms for all procedures performed (project identification code: 2297) were approved by the Ethics Committee for Human Participants of Niigata University School of Medicine (Niigata, Japan) in accordance with the Helsinki Declaration of 1964 (as revised in 2013).

**Figure 6:**
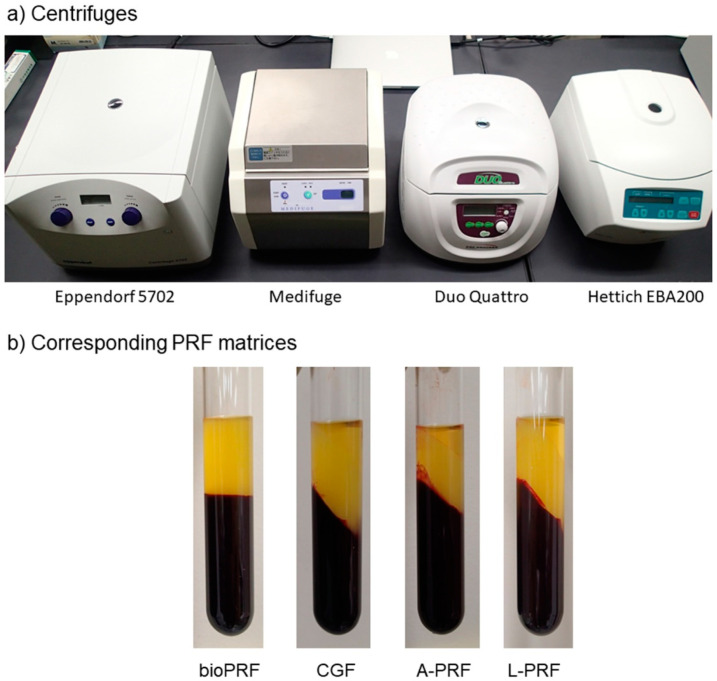
(**a**) Centrifuges used in this study. (**b**) The appearance of the corresponding PRF matrices in glass tubes after centrifugation.

As described previously [11], blood (~9 mL) was collected from the volunteers in plastic vacuum plain blood collection tubes (Neotube #NP-PN0909; Nipro, Osaka, Japan) by peripheral venipuncture in one of the antecubital fossa veins. The blood samples were then transferred to plain glass tubes (Nichiden-Rika Glass Co Ltd., Kobe, Japan) and immediately centrifuged under the recommended centrifugal conditions using four types of centrifuges, one horizontal centrifuge (#5702; Eppendorf, Hamburg, Germany) and three fixed angle centrifuges [Medifuge (Silfradent S. r. l., Santa Sofia, Italy), Duo Quattro (Process for PRF, Nice, France), and EBA200 (Hettich, Vlotho, Germany)] (Figure 6a) (Table 1). Depending on the protocols of the individual centrifuges, distinguishable PRF matrices were formed (Figure 6b).

A fraction of red blood cells (RBC) (e.g., clot) was cut off using scissors. Then, the PRF matrices were gently washed with phosphate buffered saline (PBS) and fixed in 10% neutralized formalin (FUJIFILM Wako Pure Chemical Corp., Osaka, Japan) for 3–5 h in plastic tubes to prevent the collapse of the original PRF matrix shape. Thereafter, the PRF matrices were transferred to PBS and stored at 4 ℃ until use, usually within 24 h.

To record the basic characteristics of individual blood samples, the number of platelets and other blood cells in the whole-blood samples and PRP preparations was determined using an automated hematology analyzer (pocH 100iV; Sysmex, Kobe, Japan).

Although several protocols called for specific tubes (e.g., silicone-coated glass tubes or silica-coated plastic tubes), the blood samples were initially collected in plain plastic tubes, then transferred into plain glass tubes within a short period of time (~2 min) [11]. It should be noted that the main purpose of this procedure was to minimize variations in the results due to tube type, as well as highlight the effects of centrifugal conditions. For example, silicone used to coat the inner surface of glass tubes impedes contact between coagulation factor XII in the blood and the silanol residue on the glass surface to delay coagulation. On the contrary, silica-coated tubes facilitate coagulation before centrifugation, such that much of the red blood cell fraction is clotted, resulting in smaller PRF clots.

### 4.2. NIR Imaging of Platelet Distribution in PRF Matrices

**Figure 7:**
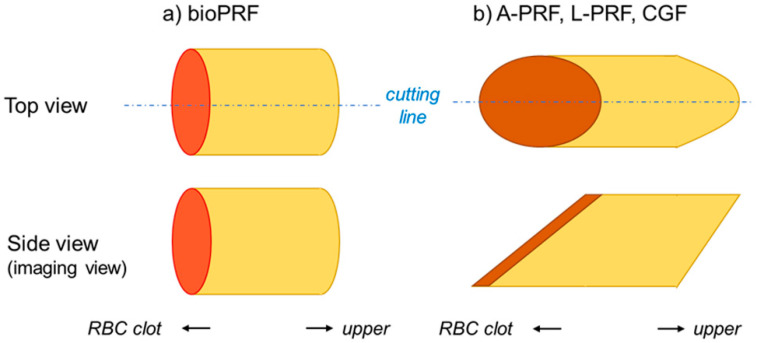
The differences in the shapes of individual platelet-rich fibrin (PRF) matrices. The shape of bio-PRF (**a**) is distinguishable from those of other PRF matrices (**b**). The “cutting line” represents the center line of PRF matrices for the sagittal section.

The fixed PRF matrices were sagittally divided into two equal pieces (Figure 7). The 3D structure of the bio-PRF matrix was cylindrical; thus, its top view was very similar to its side view. After fixation, the PRF matrix was sagittally divided into two, almost equal pieces along randomly chosen lines. The 3D structures of the A-PRF, L-PRF, and CGF matrices were also cylindrical, but they were distorted against the long axis. Thus, in the side view, their shape was a parallelogram. The PRF matrices were divided along the central axis of their top view.

Of the two PRF pieces, one was used for the detection of CD41^+^ platelets, while the other was used as an isotype control [11]. The PRF pieces were dehydrated using KimWipes (Kimberly-Clark Corp., Dallas, TX, USA) and washed twice in PBS containing 0.1% Tween 20 (0.1T-PBS) in six-well plates for 10 min using a vortex mixer. After dehydration, the PRF pieces were blocked for 24 h in 2% Block Ace (Sumitomo Dainippon Pharma, Osaka, Japan) solution dissolved in 0.1 T-PBS (BA-TPBS) in 2-mL sample tubes at 4 °C. The PRF pieces were then dehydrated, washed twice (as described above), and probed with mouse monoclonal human CD41 antibody (BioLegend, San Diego, CA, USA) at a 3:1000 dilution for 24 h at 4 °C. The PRF isotype controls were treated with non-immunized mouse IgG (BioLegend). After primary antibody treatment, the PRF pieces were dehydrated, washed in 0.1 T-PBS using a vortex mixer, and probed with NIR dye-conjugated goat anti-mouse IgG (1:4500) (iFluor 790; AAT Bioquest, Inc., Sunnyvale, CA, USA) for 90 min at 4 °C. The PRF pieces were then washed using a vortex mixer, dehydrated using KimWipes, and scanned at 800 nm using a Pearl NIR imaging system (Li-Cor, Lincoln, NE, USA). The dye was excited using an 800 nm channel laser source (solid-state laser diode at 785 nm). An emission at 820 nm was detected using a cooled CCD detector.

The total florescence intensity of each half of the PRF membrane was measured using software provided by Li-Cor. The fluorescence intensity of the background was subtracted from that of the matrix. The fluorescence intensity of the CD41 antibody-reactive proteins was expressed as the ratio of the fluorescence intensity of CD41 to that of the corresponding isotype control.

Strong correlations between fluorescence intensity and fluorescence dye concentration or suspended platelet count were reported in our previous study, where approximately 80% of near-infrared light (800 nm) was transmitted through a compressed dehydrated PRF membrane [11].

### 4.3. Immunohistochemical Examination

Freshly prepared PRF matrices were gently, but not fully, compressed using a stainless-steel PRF compression device (PRF stamper; JMR Corp. Ltd., Niigata, Japan); then, they were fixed, divided into seven pieces, dehydrated in a series of ethanol washes, embedded in paraffin, and sectioned for immunohistochemical examination using rabbit polyclonal anti-CD41 antibody (GeneTex, Irvine, CA, USA), horseradish peroxidase-conjugated goat anti-rabbit IgG antibody (Cell Signaling Technology, Danvers, MA, USA), and 3,3′-diaminobenzidine (DAB) substrate solution (Kirkegaard & Perry Laboratories, Inc., Gaithersburg, MD, USA) [7,12].

### 4.4. Statistical Analysis

The data are presented as box plots (Figure 2) and expressed as the mean ± standard deviation (Figure 3). For the fluorescence intensity data in Figure 2a, SigmaPlot (SigmaPlot 13.0; Systat Software, Inc., San Jose, CA, USA) was used to evaluate the image analysis data as being parametric by both normality (Shapiro–Wilk) and equal variance testing. One-way analysis of variance (ANOVA) was followed by Bonferroni’s multiple-comparisons test. For the length data in Figure 2b, the normality test failed. As such, a non-parametric Kruskal–Wallis one-way ANOVA on ranks was performed. All pairwise multiple comparisons were tested using Tukey’s test. For the comparison of the three parts in Figure 3, a non-parametric Kruskal–Wallis one-way ANOVA on ranks was performed, but no subsequent multiple-comparisons tests were performed. *p* < 0.05 was considered statistically significant.

## 5. Conclusions

In PRF matrices, platelet distribution depends to a great extent on the centrifugal force and type of rotor used, rather than on the specific gravity of the platelets. In this study, a buffy coat-like fraction did not form on the interface between the RBC fraction and the plasma/PRF fraction. When an angle rotor was used, an increased centrifugal speed was found to alter the distribution of platelets, changing from a widespread, homogenous distribution to an accumulation of platelets on and below the distal surface of the PRF matrices. However, various types of blood collection tubes are used in clinical settings. Compared with glass tubes, silica-coated plastic tubes facilitate coagulation and distribute platelets widely and homogenously in the resulting PRF matrices, regardless of the centrifugal speed used [12], while silicone-coated glass tubes delay coagulation, an effect which is expected to modify platelet distribution by interfering with the contact of coagulation factor XII with the glass surface. Thus, we should be careful with the devices used. 

## Figures and Tables

**Table 1:** Devices and centrifugal conditions for preparation of individual PRF matrices.

Title	Bio-PRF	CGF	A-PRF	L-PRF
Vacuum tubes (manufacturer)	Plain plastic tube (Nipro)	Plain plastic tube (Nipro)	Plain plastic tube (Nipro)	Plain plastic tube (Nipro)
2nd tubes (manufacturer)	Plain glass tube (Nichiden-Rika Glass)	Plain glass tube (Nichiden-Rika Glass)	Plain glass tube (Nichiden-Rika Glass)	Plain glass tube (Nichiden-Rika Glass)
Centrifuges (manufacture)	#5702 (Eppendorf)	Medifuge (Silfradent)	Duo Quattro (Process for PRF)	EBA200 (Hettich)
Rotor types	Swing	Angle	Angle	Angle
Rotor angulation	Horizontal	33°	41.3°	33°
Centrifugal force (time)	700× *g* (8 min)	692× *g* (2 min) 547× *g* (4 min) 592× *g* (4 min) 885× *g* (3 min)	200× *g* (14 min*)	400× *g* (12 min)

## Abbreviations

PRF	platelet-rich fibrin
A-PRF	advanced platelet-rich fibrin
CGF	concentrated growth factors
L-PRF	leukocyte- and platelet-rich fibrin
NIR	near infrared
SEM	scanning electron microcopy
3D	three dimensional
0.1T-PBS	PBS containing 0.1% Tween 20
BA-TPBS	2% Block Ace solution dissolved in 0.1% T-PBS
DAB	3,3′-diaminobenzidine
ANOVA	analysis of variance
TGFβ1	transforming growth factor β1
PDGF-BB	platelet derived growth factor-BB
VEGF	vascular endothelial growth factor
RBC	red blood cell
